# Approaches to Inactivating Aflatoxins—A Review and Challenges

**DOI:** 10.3390/ijms222413322

**Published:** 2021-12-11

**Authors:** Kinga Kutasi, Nina Recek, Rok Zaplotnik, Miran Mozetič, Mitja Krajnc, Peter Gselman, Gregor Primc

**Affiliations:** 1 Department of Surface Engineering, Jozef Stefan Institute, Jamova Cesta 39, 1000 Ljubljana, Slovenia; kutasi.kinga@wigner.hu (K.K.); nina.recek@ijs.si (N.R.); rok.zaplotnik@ijs.si (R.Z.); miran.mozetic@ijs.si (M.M.); 2 Žipo Ltd., Šetarova 21, 2230 Lenart, Slovenia; mitja.krajnc@kgskrajnc.si; 3 Interkorn Ltd., Gančani 94, 9231 Beltinci, Slovenia; peter.gselman@interkorn.si

**Keywords:** mycotoxin, decontamination, gas-discharge plasma, ozonation, ammoniation, UV

## Abstract

According to the World Health Organization, the contamination of crops with aflatoxins poses a significant economic burden, estimated to affect 25% of global food crops. In the event that the contaminated food is processed, aflatoxins enter the general food supply and can cause serious diseases. Aflatoxins are distributed unevenly in food or feedstock, making eradicating them both a scientific and a technological challenge. Cooking, freezing, or pressurizing have little effect on aflatoxins. While chemical methods degrade toxins on the surface of contaminated food, the destruction inside entails a slow process. Physical techniques, such as irradiation with ultraviolet photons, pulses of extensive white radiation, and gaseous plasma, are promising; yet, the exact mechanisms concerning how these techniques degrade aflatoxins require further study. Correlations between the efficiency of such degradation and the processing parameters used by various authors are presented in this review. The lack of appropriate guidance while interpreting the observed results is a huge scientific challenge.

## 1. Introduction

Whilst growing, field crops such as corn (*Zea mays* L.) are often exposed to pathogens (e.g., bacteria, fungi, viruses, and phytoplasmas), which can negatively impact both the yield and the quality. Ensuring a healthy population means it is necessary to be careful when fungi cause plant diseases. The saprophytic fungi that inhabit infected plant tissues can produce harmful substances known as mycotoxins.

Mycotoxins are secondary metabolites of filamentous fungi, dangerous to both humans and animals [1,2]. The most common mycotoxin-producing genera are *Aspergillus*, *Fusarium*, and *Penicillium*. The family of toxins produced by the fungi *Aspergillus flavus* and *Aspergillius parasiticus* pose the greatest danger to animal and human health. *Aspergillus flavus* is considered to be the most frequent source of aflatoxins in crops. As seed-inhabiting fungi, *Aspergillus parasiticus* can contaminate a wide variety of crops, either before harvesting in the field or after harvest while being handled and processed. Naturally occurring aflatoxins are the aflatoxin B_1_ (AFB_1_), the most potent carcinogenic agent known, and the aflatoxins B_2_, G_1_, and G_2_ (aflatoxins B_2_ and G_2_ are dihydroxylated derivatives of B_1_ and G_1_) [3]. Aflatoxins can enter the human food chain directly through the consumption of infected crops/cereals, oilseeds, nuts, or spices or indirectly, when infected crops are fed to animals. The toxins can also be transferred into animal products such as milk, eggs, and meat. For example, the aflatoxins M_1_ and M_2_, which are hydroxylated derivatives of B_1_ and B_2_, can be found in the milk of cows fed with contaminated feed [4,5]. Aflatoxins were found to increase the risk of several diseases, such as liver and lung cancer with exposure to AFB_1_-contaminated grain dust and gastrointestinal cancer following the consumption of contaminated food [2]. Aflatoxins may be present in various food and feed crops, such as corn, wheat, rice, peanuts, Brazil nuts, pistachio nuts, red chili, peppers, almonds, cattle feed, poultry feed, and many more [6].

## 2. Characteristics of Aflatoxins and Detoxification Methods

### 2.1. Toxicity and Properties

The extent of the mycotoxins’ toxicity and carcinogenicity is determined by the metabolites the mycotoxins are converted into, which can bind to critical cellular macromolecules. Aflatoxins are a family of compounds generally classified as difuranocoumarins. Inactive in their basic form, aflatoxins become carcinogenic upon biotransformation, producing metabolites capable of reacting with macromolecules. The metabolism of aflatoxins and the methods to modulate the metabolism are presented by Guengerich et al. [7]. Aflatoxin derivatives covalently bind to macromolecules through the unsaturated furan ring, leading to epoxidation (i.e., oxidation of the furan ring). AFB_1_ is converted into the reactive 8,9-exo-epoxide and 8,9-endo-epoxide, with the exo-epoxide being the toxic species responsible for the AFB_1_ genotoxic properties [8], which bind to deoxyribonucleic acid (DNA) to form the predominant 8,9-dihydro-8- (N7-guanyl)-9-hydroxy-AFB_1_ (AFB_1_-N7-Gua) adduct [9], as also detailed in [7]. Upon the adduct forming, the cell starts to become a tumor cell. It was also suggested that AFB_1_ induces reactive oxygen species (ROS) and thus oxidative stress, which activates mitochondrial ROS-dependent signal pathways, inducing apoptosis through the mitochondrial signal pathway [10].

**Figure 1:**
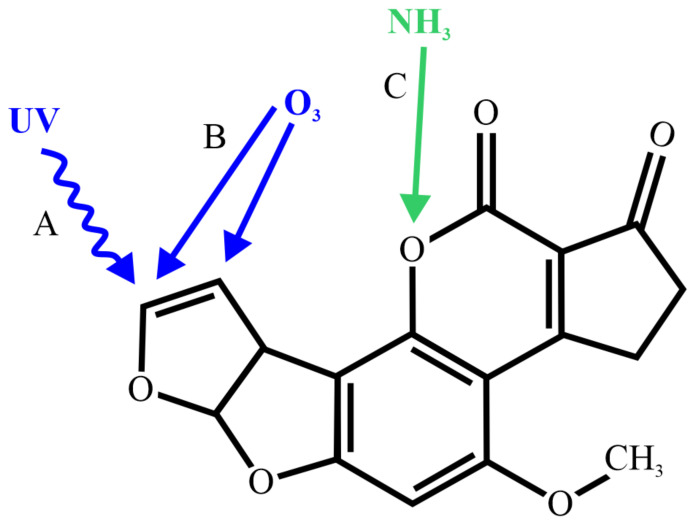
Schematic of AFB_1_ showing the sites of reactivity for three inactivation methods: (**A**) UV light; (**B**) ozone; and (**C**) ammonia. For each inactivation method, the reactivity site is specifically marked.

The properties of aflatoxins were reviewed by Quadri et al. [11]. Aflatoxins are slightly soluble in water, insoluble in non-polar solvents, and freely soluble in polar organic solvents. They have strong thermal stability and remain stable even at temperatures above 100 °C, preventing them from thermally degrading during food manufacturing, such as while pasteurizing milk and dairy products [5,12]. Significant decontamination was reported only when heating or roasting at temperatures of 150 °C or higher [13]. Aflatoxins are unstable in ultraviolet (UV) light in the presence of oxygen, in extreme pH conditions, such as pH < 3 or >10, and in the presence of oxidizing agents. In addition, the lactone ring of the coumarin structure is susceptible to alkaline hydrolysis, ammonia, and hypochlorite. These properties have led to the development of different decontamination methods, depicted schematically in Figure 1, which can significantly reduce the amount of mycotoxins in seeds and crops [14].

### 2.2. UV Radiation and Pulsed Light Treatment

**Figure 2:**
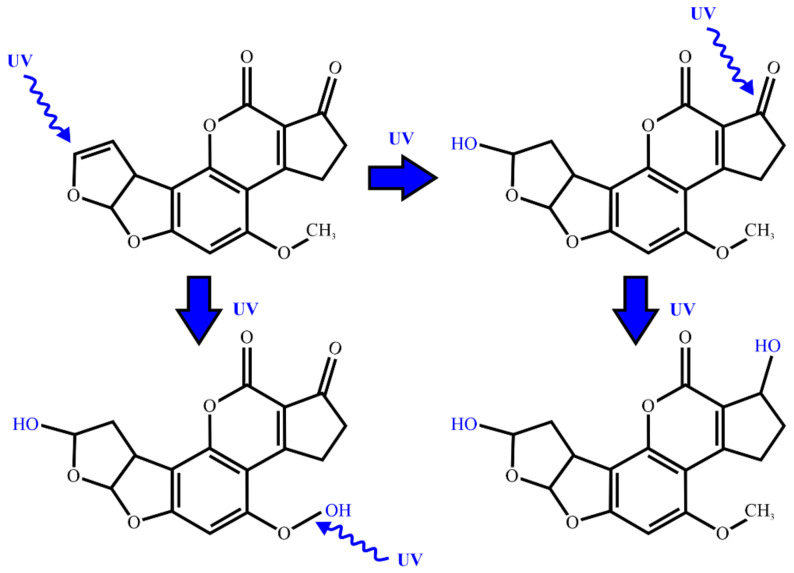
Mechanism of reaction with UV light; aflatoxin AFB_1_ reacts with photons of UV light in water (aqueous medium): 2 different degradation products are formed. Adapted from [16].

The study of light-induced mycotoxin decontamination dates back to the 1980s [15] and recently includes more thorough investigations. The photo-degradation of aflatoxin B_1_ is schematically presented in Figure 2 and was recently studied in a water solution by Liu et al. [16]. Water solutions of various AFB_1_ concentrations were irradiated at 4 °C with an ultraviolet lamp using different intensities. Total degradation of AFB_1_ was obtained with treatments of 60 min, 80 min, and 100 min while applying intensities of 800 µW/cm^2^, 400 µW/cm^2^, and 200 µW/cm^2^, respectively. Three degradation products were identified, i.e., C_17_H_14_O_7_, C_16_H_14_O_6_, and C_16_H_12_O_7_, using ultra-performance liquid chromatography-quadrupole time-of-flight mass spectrometry (UPLC-Q-TOF MS). Similar studies were conducted by Patras et al. [17] who used the collimated 200–360 nm beam of a 1000 W medium-pressure UV lamp to irradiate water solutions with different aflatoxin concentrations. Irradiation doses of 4.88 J/cm^2^ (meaning a 40 min treatment) were shown to reduce the aflatoxin concentrations as follows: 67% for aflatoxin G_1_, 30% for aflatoxin B_2_, and 98% for aflatoxin B_1_. It was hypothesized that the aflatoxin degradation occurs through photolysis due to the OH radicals created by the ultraviolet (UV) radiation in the liquid media. The HepG2 in vitro cell culture model was used to assess the UV-irradiated aflatoxin’s toxicity. It was demonstrated that increasing the UV dosage decreased the cytotoxicity caused by aflatoxins in the cells.

Atalla et al. [18] investigated the effect of UV (254 nm and 362 nm) and fluorescent light (fluorescent tube 30 W) on the mycotoxin production of different fungi grown on wheat grains. The wheat grains were artificially inoculated and treated at a distance of 5 cm and 25 cm from the UV and fluorescent lamps. After light treatment, the grains were stored for three weeks under different relative humidities: 50%, 74%, and 80% at room temperature. It was found that a 30–60 min treatment completely halted the aflatoxin production of *A. parasiticus*. Shanakhat et al. [19] showed that UV irradiation of 15 min of aflatoxin-contaminated semolina could lead to the complete degradation of AFB_1_. A germicidal UV lamp with 30 W power providing UV-C radiation at 254 nm was used as a light source, and 100 g of the semolina layer of 1 cm thickness was exposed to the UV light at a distance of 15 cm. The initial AFB_1_ concentration was 1.2 µg/kg.

The reduction in *Aspergillus* spp. counts and aflatoxin production by a 254 nm UV-C treatment was investigated on artificially inoculated hazelnuts by Basaran et al. [20]. With a 6 h (2 h periods repeated 3 times) UV-C (9.99 J/cm^2^) treatment, a 2-log reduction in *Aspergillus* spp. counts and a 25% reduction in aflatoxins B_1_ and G_1_ were obtained. No changes in the macromolecular components of the hazelnuts due to the UV-C treatment were detected. Decontamination of infected nuts was also investigated by Jubeen et al. [21]. The moisture content of randomly selected nuts (ground and tree) was artificially increased to facilitate mold growth during 12 weeks of storage at 25–30 °C. The fungal spores detected on the seeds immediately after selection belonged to *Aspergillus flavus*, *Aspergillus parasiticus*, and *Penicillium*. Inside transparent 1 cm thick polythene pouches, 200–250 g nuts were packed and then placed at a distance of 25 cm from a 265 nm UV source of 108 J/m^2^. Treatments were performed for up to 45 min at room temperature. The fungicidal activity of UV-C radiation was found to be more pronounced in the nuts treated at the higher 16% moisture level, with levels of efficiency in the following order: walnut > almond = pistachio > peanuts. A 45 min treatment resulted in 87% and 96% degradation of AFB_1_, depending on the moisture level, with the maximum reduction seen for almond and pistachio. The highest initial AFB_1_ level of 158.68 µg/kg was found in the peanuts, with a 16% moisture level, which also could be reduced by 96% with a 45 min treatment.

Another decontamination technique, supposedly based on UV radiation, is the pulsed light technique, which, compared to UV-lamps, has the advantages of a broad UV spectrum, a short duration, but high peak power. Moreau et al. [22] evaluated the effectiveness of pulsed light technology for degrading mycotoxins in a water solution. The treatment chamber was equipped with four orthogonally positioned xenon lamps (radiating in the 180–1100 nm wavelength range), which generated a light flux of 1 J/cm^2^ during a single 300 µs flash. Furthermore, 2 mL of mycotoxins (in the case of AFB_1_ of 5 µg/mL concentration) was exposed to one to eight flashes of pulsed light. It was found that eight flashes destroyed 84%, 72%, 92%, and 98% of the zearalenone, deoxynivalenol, aflatoxin B_1_, and ochratoxin in the solution. By evaluating the genotoxicity of the mycotoxins, it was shown that the treatment of zearalenone and deoxynivalenol by single or multiple flashes of pulsed light marginally reduced their toxicity. In contrast, in the case of AFB_1_, pulsed light could completely eliminate its mutagenic potential. Similar studies with a similar xenon lamp were conducted by Wang et al. [23], investigating the effect of pulsed light on the degradation of AFB_1_ and AFB_2_ in rice bran and rough rice. Rough rice was inoculated with *Aspergillus flavus* and allowed to grow for 14 days to produce AFB_1_ and AFB_2_. Fifty grams of inoculated rough rice was spread uniformly in a single layer and treated for 20 s to 80 s in 20 s periods with 0.52 J/cm^2^ pulses at room temperature. In the case of rice bran, a 3 g sample was spread uniformly with a thickness of 1 mm and treated in 5 s periods for up to 15 s. It was found that the 80 s treatment of the contaminated rough rice could reduce the AFB_1_ and AFB_2_ levels by 75.0% and 39.2%, respectively, while the 15 s treatment of the rice bran reduced the levels by 90.3% and 86.7%, respectively. The genotoxicity tests showed that after treatment, the AFB_1_, AFB_2_, and their residual by-products had no mutagenic activity, while the toxicity of the two aflatoxins was significantly reduced.

### 2.3. Ammoniation

**Figure 3:**
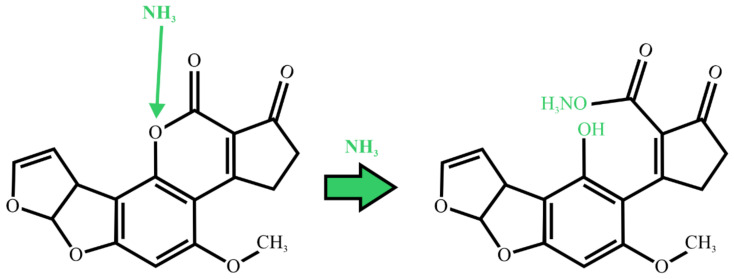
Mechanism of reaction of ammoniation of AFB_1_. Adapted from Stanley et al. [26].

One method for aflatoxin deactivation in maize that is approved in several countries is ammoniation. The ammoniation process uses either ammonium hydroxide or gaseous ammonia. It can be utilized in two ways: a high-pressure and high-temperature (HP/HT) process or an atmospheric pressure and ambient temperature process (AP/AT). The HP/HT process involves treating the contaminated product with anhydrous ammonia and water in a contained vessel, with the amount of ammonia being 0.5–2%, the moisture 12–16%, the pressure 2.4–3.8 bar, the treatment time 20–60 min, and the temperature 80–120 °C. The AP/AT process also uses anhydrous ammonia and water sprayed on the seed as it is packed into a plastic silage-type bag. As the temperature and pressure are much lower, the processes can take 12–42 days. Park et al. [24] reviewed the reduction in aflatoxin hazards by ammoniation. The chemical effect of ammonia on aflatoxin inactivation is reported to start upon the opening of the lactone ring of AFB_1_ and is followed by ammonium salt forming from the resulting hydroxy acid (Figure 3). The reversion of reaction products back to the parent AFB_1_ was observed under acidic conditions when the exposure time and process conditions were insufficient [25].

The aflatoxin/ammonia reaction has several products, their relative amounts being dependent on the temperature and pressure conditions used and whether ammonium hydroxide or ammonia gas is the ammonia source [25,26,27,28]. These products have been found to hold a relative mutagenic/toxic potential (the covalent bonding to the DNA) that is 20- to 3000-fold less than the parent aflatoxin [25]. The efficiency and the irreversibility of the ammoniation process on naturally contaminated corn were demonstrated by Weng et al. [29], who treated 50 g of naturally contaminated, ground yellow corn containing 7500 ng/g of AFB_1_ for 60 min in several different conditions with respect to the ammonia concentration, moisture level (12% and 16%) of the corn, temperature, and applied pressure. The treatment with 2% gaseous NH_3_ at 40–45 °C and 3.8 bar reduced the AFB_1_ level in samples containing 12% moisture by 15% (from 67.7% to 52.7%), while in the samples containing 16% moisture the reduction changed from 93.1% to 79.4%, i.e., by 14%. The treatment with aqueous NH_4_OH alone at 121 °C and 1.2 bar, or followed by gaseous NH_3_ treatment, reduced the AFB_1_ content by more than 99%. Reversibility was found to be less than 0.05%. Corn was also treated in an industrial-scale mobile ammonia treatment plant capable of processing 50 metric tons of whole kernel corn per hour. Over 30,000 metric tons of corn containing aflatoxin levels ranging from 100 to 500 µg/kg was successfully treated to reduce the aflatoxin levels to below 20 µg/kg.

Gomaa et al. [30] treated artificially infected corn with a final 4000 µg/kg aflatoxin contamination with AP/AT or HP/HT (high pressure of 2 bar and temperature of 121 °C) methods. The moisture content of the corn was adjusted to an 18% wet basis and sprayed to provide levels of 0.25% to 2% ammonia on a dry-matter basis. Polyethylene bags were filled with 1 kg of contaminated corn and treated under AP/AT or HP/HT conditions for 24 h. It was found that the efficiency of destroying the aflatoxins increased with a higher ammonia concentration. At atmospheric pressure (AP/AT), 90% total aflatoxin destruction was obtained with 2.0% ammonia, while at high pressure (HP/HT) the destruction exceeded 99%. With the AP/AT method, the highest 99% destruction was reported by Norred et al. [31] on 1000 µg/kg of naturally aflatoxin-contaminated corn.

### 2.4. Ozonation

**Figure 4:**
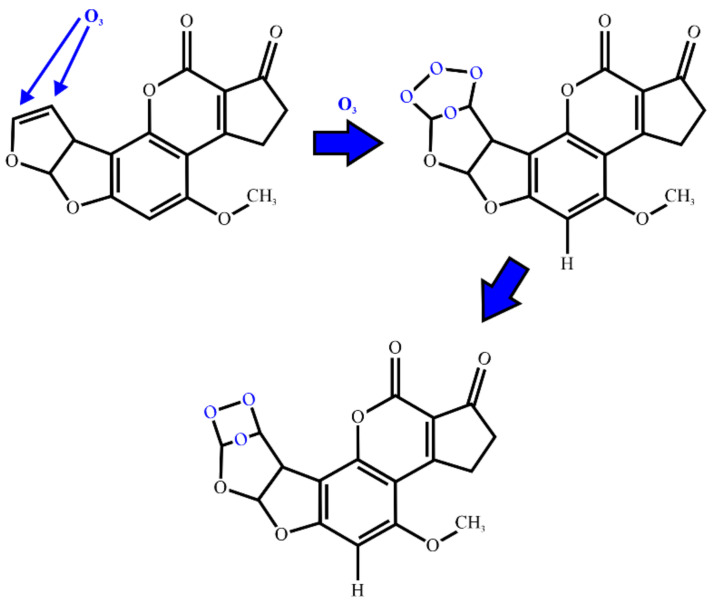
The reaction mechanism of AFB_1_ with ozone, O_3_. Degradation leads to aflatoxin molozonide and further, spontaneously, to aflatoxin ozonide. Adapted from [6].

Another technique still attracting considerable research attention is the use of ozone (both aqueous and gaseous ozone) to reduce fungal and mycotoxin contamination. The different ozone-generation methods, such as water electrolysis or electrical gas discharge, by allowing the ozone production on-site from ambient air, offered a significant advantage over other techniques that require costly production, storage, and transportation. Ozone decomposes into atomic and molecular oxygen, meaning that in this process there are no residues of toxic chemicals, no danger of chemical-mixing hazards, and no need to specially treat the exhaust. A suggested initial stage in the transformation of AFB_1_ upon ozonation is presented in Figure 4.

While the ammoniation opens the lactone ring in the coumarin, ozone attacks the C8-C9 double bond on the terminal furan responsible for the mutagenicity and carcinogenicity of the aflatoxins [8], which causes them to break down into organic acids, aldehydes, ketones, and carbon dioxide [32,33]. The effect of ozone on naturally contaminated corn with AFB_1_ was tested by McKenzie et al. [32]. Corn contaminated with 1220 µg/kg AFB_1_ was treated for 92 h with 14.0 wt% O_3_ at a flow rate of 200 mg/min in 30 kg batches, which led to an AFB_1_ reduction of over 95%. The corn’s toxicity was tested on turkeys by feeding them with clean (control), treated control, untreated, and treated AFB_1_ corn, respectively. Compared with the controls, the turkeys fed the AFB_1_ corn gained less body and relative liver weight, whereas the turkeys fed the ozone-treated control or ozone-treated AFB_1_ corn did not differ from the controls. Furthermore, alterations in the majority of the relative organ weights, liver discoloration, serum enzyme activity, hematological parameters, and blood chemistry caused by AFB_1_ were eliminated by the ozone treatment [32].

Luo et al. [34] examined the effect of ozone treatment on degrading AFB_1_ in artificially infected maize. They evaluated the toxicity of the degradation products using the human hepatocellular carcinoma cell line (HepG2) as model cells. Corns of different moisture content were treated in 10 g batches in a 1 L glass reactor in flowing gas with ozone concentrations ranging from 40–90 mg/L for up to 40 min at 25 °C and 75% relative humidity. The results showed that the degradation was more efficient in the lower 13.47% of the moisture content of the corn, and the 20 min and 40 min treatment with 90 mg/L ozone led to decreases of 83 µg/kg AFB_1_ to 18.12 µg/kg and 9.9 µg/kg, respectively. The toxicity test showed the AFB_1_-contaminated corn extract had high cell toxicity, while the ozone-treated infected corn had no significant effect, similar to the AFB_1_-free culture solution.

Prudente et al. [35] demonstrated the effect of ozonation on naturally contaminated corn. Contaminated and non-contaminated corn seeds were treated in 113 L containers with 10 to 12 wt% ozone, flowing at a flow rate of 2 L/min, for 96 h at 12 to 15 h intervals, with mixing occurring every 30 h. This produced a 92% reduction in AFB_1_, with the AFB_1_ level decreasing from 586.8 µg/kg to 47.7 µg/kg, while no reversion of inactivated aflatoxin to the parent compound was observed. The AFB_1_ could be further reduced to about 29 µg/kg through exposure to an acidic environment in the ozone-treated corn. It had previously been shown that the acid treatment led to hydration of AFB_1_ at the 8,9-olefinic bond of the terminal furan ring to form aflatoxin B_2a_, whose toxicity was less than 1/200 that of AFB_1_ [14]. It was also found that the ozonation of clean corn had no significant effect on the saturated and unsaturated fatty acids. In contrast, in contaminated corn, the percentage of unsaturated fatty acid was significantly reduced from 82.0% to 79.1%, whereas the saturated fatty acid composition rose from 18.0% to 20.9%. While evaluating the mutagenicity of corn extract, it was found that the ozonation procedure reduced the mutagenic potential of the AFB_1_-contaminated corn, suggesting that the ozonation process may have caused the formation of fat-soluble reaction products that hold relatively low mutagenic potential. It was also hypothesized that the ozonation decontamination process might have produced oxidation products such as oxidized linoleic acid, malonaldehyde, and acrolein that have been shown to be mutagenic in the Salmonella mutagenicity assay [35].

Using infected peanut kernel, Proctor et al. [36] showed that aflatoxins B_1_ and G_1_ are more sensitive to ozone and heat treatment than B_2_ and G_2_, which is significant given that aflatoxins B_1_ and G_1_ are the most potent toxins known. However, the highest level of degradation of AFB_1_ (77%) was obtained when the ozone treatment was performed at an elevated temperature of 75 °C.

The inhibitory effect of ozone on the growth of *Aspergillus flavus* and the AFB_1_ production were investigated by Ta et al. [37] in vitro, using culture media. They showed that 40 ppm ozonation inhibited the mycelial growth of fungi by 65% and 95% for the 5 min and 20 min treatments, respectively. Moreover, the AFB_1_ production also decreased, while in the untreated sample the AFB_1_ concentration was 185 µg/100 mL; in the 20 min treated sample, it was only 42 µg/100 mL. These results infer that, with ozonation, the growth of fungi can be stopped (or they can even be killed) prior to the grains being stored, inhibiting the production of AFB_1_.

The effect of ozone on seed germination, which is an important factor if crops are meant to be used as seeds, was investigated for barley by Allen et al. [38] using different ozone doses. It was established that ozone doses of less than 0.98 mg/(g barley)/min showed no effect on barley germination even after 45 min of ozonation. When using an ozone dose of 0.98 mg/(g barley)/min, at ozonation times longer than 10 min, the germination started to decrease and saw a reduction of 30% after a 45 min treatment. A similar test was conducted on wheat by Savi et al. [39], showing that with a 120 min exposure to 60 µmol/mol O_3_ wheat germination was not affected, while a 180 min exposure reduced the germination capacity by 12.5%. In addition, at both treatment times, no modifications in the length of the coleoptile or the seminal root of the germinated wheat seeds were observed. They also tested the penetration efficiency of ozone into the kernel. Deoxynivalenol (DON) injected by syringe into the grain endosperm was found to have been reduced to the limit of detection after 180 min of treatment.

Kells et al. [40] intended to optimize the ozone flow in a 12.7-tonne capacity, 3 m diameter galvanized steel grain bin where ozone was forced downward through the grain to exit the plenum. They treated 8.9 metric tons of maize with 50 ppm ozone for 3 d, which led to a 92–100% mortality of several insects and a 63% reduction in fungus contamination of the kernel surface. Two distinct phases were identified in the penetration and distribution of ozone in the system. The first phase was characterized by the ozone’s rapid degradation and slow movement through the grain until the active sites became saturated, which depended on the gas flow velocity. In contrast, in the second phase, the ozone flowed freely through the grain with little degradation. For efficient fumigation, the second phase should be achieved while the time for the first phase should be minimized. The generator used in the study produced 50 ppm of ozone at an apparent velocity of 0.0030–0.0036 m/s. Ozone was detected within 0.5 d at a depth of 0.3 m in the center, 1 d at 0.9 m in the center, 12 d at 1.5 m in the center, 18 d at 1.8 m in the center, 8 d at 1.5 m from the southern wall, and after 13 d at 1.8 m from the southern wall of the bin. The ozone levels stabilized at 20 d; however, the first phase was not completed. In the case of a much smaller, 0.57 m diameter laboratory reactor, an optimum gas velocity of 0.03 m/s was determined, which allowed the deep penetration of ozone into the grain mass, namely 85% of the ozone penetrated 2.7 m into the column of the grain in 0.8 d during the first phase.

It was demonstrated that ozonation could efficiently reduce the grain’s surface contamination with microorganisms, insects, and mycotoxins; yet, long treatment times are needed, which in the case of industrial-scale storage bins can take weeks. A disadvantage of this long treatment is that the high ozone concentration required for decontamination can also induce noticeable damage to the equipment through corrosion. On the other hand, in kernels naturally contaminated with mycotoxins, the achieved decontamination does not reach the safety level due to the ozone’s low penetration efficiency into the kernel. An alternative to ozonation and ammoniation could be gas discharge plasmas, rich in reactive oxygen and nitrogen species, UV radiation and energetic particles, which provide the synergy of the oxidative species and UV radiation and may also enhance the penetration of oxidative species.

## 3. Characteristics of Aflatoxins and Detoxification Methods

### 3.1. Relevance of Non-Thermal Gas Discharge Plasmas for Agriculture

Gas discharge plasmas are weakly ionized gases generated from neutral gases by energy input. The energy is preferentially transferred to the electrons, due to which the neutral species’ temperature, and thus the gas temperature, remains low, close to room temperature, while the electrons gain high energies that make them able to ionize, excite, and dissociate the background gas molecules. In plasmas generated from nitrogen and oxygen-containing gas mixtures with a certain humidity, reactive oxygen and nitrogen atoms, reactive metastable oxygen molecules, ozone, OH and NO radicals, and UV-radiating excited species can be created, which opens up the field of biological applications of gas discharges.

In general, low-pressure discharges can be generated in large volumes. The most common discharge systems relevant to large-scale applications are the direct current and capacitively coupled radio-frequency discharges, which are generated between two electrodes [41,42,43], electrodeless inductively coupled radio frequency discharges [43,44,45], and surface-wave microwave discharges [46]. The active species generated in smaller volume discharges can also be transported by gas flow into larger volume reactors called plasma afterglows [44,45]. In low-pressure systems, the samples should be placed in a vacuum, which may prove to be a limiting factor for some biological samples. On the other hand, at atmospheric pressure, where no vacuum system is needed, the size of the plasma is limited to a few millimeters in the vicinity of the discharge electrode due to the very short mean free path length of energetic electrons [47]. The atmospheric pressure systems of relevance to industrial applications are dielectric barrier discharges (DBD) formed in the narrow gaps between closely spaced electrodes, at least one of which is covered by a dielectric [48], the surface dielectric barrier discharges (SDBD) [49,50], and plasma jets, where the applied zone is the afterglow similar to the low-pressure case [51].

Research on the applicability of discharge plasmas to biological systems has boomed in the last two decades. At the beginning, studies focused on medical tools and wound sterilization (bacteria, virus, and protein inactivation), suggesting different low-pressure [45,49,52] and non-thermal high-pressure discharge systems [53,54]. The success of non-thermal discharge plasmas in sterilization led to broader applications, such as in agriculture and food processing [52,55]. In the field of agriculture, the research first focused on improving seed germination and plant growth and cleaning from pathogens using gas phase plasma species, such as OH, O_3_ and NO molecules, O-atoms, and UV radiation [56,57]. In the case of seed germination, it is generally accepted that ROS and NO are crucial for breaking the dormancy of the seeds [58,59].

Low-pressure systems were used to test the effect of plasma species on seed germination and to reduce seed-surface contamination. Barley and corn seeds were treated with a direct current (DC) low-pressure discharge in the residual atmosphere at 15 Pa. It was found that the fungal load could be significantly reduced with a 20 min treatment, while the germination rate of barley decreased by 12% and that of corn by 5% [60]. It was established that the efficiency of the treatment depends on the type of seed, size, and surface properties. At a higher pressure of 150 Pa, wheat seeds were treated with helium capacitively coupled radio-frequency discharge (CCP) with an electrode distance of 2 cm for 15 s. This treatment was found to increase the germination and growth rate and the production yield [61]. We note that here the presence of reactive oxygen and nitrogen species is minimal. On the contrary, when using the afterglow of an air microwave discharge (rich in reactive species) at the same pressure (140 Pa, 500 W), it was established that treatment times of 180−2400 s led to decreased wheat germination and had no significant effect on the oat seeds [62].

Szőke et al. [63], using the flowing afterglow of a low-pressure Ar/N_2_/O_2_ surface-wave microwave discharge (abundant in O and/or N atoms, O_2_(a) and NO molecules, and UV radiation), studied the effect of the ROS and reactive nitrogen species (RNS) on barley and corn seed germination, and their surface disinfection from two types of *Fusarium*: *graminearum* and *verticillioides*. It was shown that the germination and vigor of the non-infected seeds were not significantly affected when barley was treated for 120 s at 200 Pa and corn for 240 s at 400 Pa. On the other hand, the seeds could be decontaminated from the germination inhibitors *Fusarium graminearum* and *Fusarium verticillioides*, namely when barley was treated for 3 min at 400 Pa in the afterglow of the mixture of 80% Ar and 20% O_2_ discharge and corn for 4 min at 800 Pa in the mixture of 80% Ar and 20% O_2_, followed by a 2 min treatment in the N_2_-2%O_2_ afterglow. These treatments also increased the germination of the infected seeds from 35% and 50%, respectively, to above 80%. It was also found that high NO content mixtures and the heating of the seed surface by the recombination of O and N-atoms inhibited barley germination. Zahoranova et al. [64] used an SDBD in ambient air in atmospheric pressure conditions. They showed that a 90 s treatment of artificially infected wheat seeds could completely inactivate *Fusarium nivale* and *Fusarium culmorum*, while for *Aspergillus flavus* 240 s was needed. However, the results showed that the seeds did not germinate after treatment. These results reveal that fine-tuning of the plasma species is needed to decontaminate seeds without inhibiting their germination.

### 3.2. Degradation of Mycotoxins by Discharge Plasmas

In the last two decades, comprehensive work has been undertaken to study the interaction of plasma species with bacteria, bacteria spores, and prions (infectious proteins). The microbial inactivation ability of the discharge plasmas was generally attributed to the oxidizing species, such as O-atoms, singlet metastable O_2_(a), O_3_, OH, the energetic ions, and UV radiation. The O-atoms can etch the organic material, thereby contributing to the volatilization of the proteins and the protective cell wall of bacteria and spores [65]. A similar role can also be attributed to the other oxygen species. Furthermore, energetic ions in the plasma environment can enhance the etching efficiency as a synergetic effect of the physical sputtering by ions and chemical etching by oxygen species [66]. Once the integrity of the cell wall is damaged, the UV radiation can penetrate the cell, reach the DNA, and induce strand breaks, thus stopping the cells from replicating [67]. It was also suggested that electrostatic forces could cause rupture of the cell membrane and, subsequently, cell death due to the accumulation of charged species on the outer cell membrane [53]. Based on these studies, the investigations were extended to mycotoxins.

There are several methods for detecting and quantifying aflatoxins in foods, including thin-layer chromatography (TLC), high-performance liquid chromatography (HPLC), mass spectroscopy (MS), enzyme-linked immune-sorbent assay (ELISA), and electrochemical immunosensor. Each of these methods has its advantages and limitations in aflatoxins analysis [68]. The degradation of different mycotoxins by gas discharge plasmas was studied by Park et al. using an argon atmospheric pressure microwave discharge [69]. The discharge was generated at the end of a quartz nozzle using a gas flow rate of 100 l/min. The intensity of UV light radiated by the plasma was detected to be in the range of 75−102 mW/cm^2^ at a wavelength of 254 nm. For the degradation test, these mycotoxins were dissolved in chloroform, and afterwards, the suspensions were inoculated on glass slides and allowed to dry at room temperature. The inoculated glass slides were placed in front of a nozzle and treated for 1, 3, 5, and 10 s, respectively. After plasma treatment, the samples were recovered from the glass slides by dissolution. These extracts were used to determine the mycotoxin concentration and cytotoxicity of the reaction products on the cell cultures. It was found that the concentrations of aflatoxin B_1_, deoxynivalenol, and nivalenol (NIV) had fallen below the detection limit after the 5 s plasma treatment. It was concluded that the degradation and the removal of mycotoxins were due to the UV irradiation and the etching by the plasma species. It was also shown that while the standard AFB_1_, DON, and NIV resulted in a significant dose-dependent decrease in the viability of mouse macrophage cells, implying their high cytotoxicity to mammalian cells even at micromolar concentrations, the plasma treatment reduced the viability loss of macrophages; the mycotoxin-induced cytotoxicity was completely prevented after 5 s of incubation.

The degradation of AFB_1_ by a low-pressure discharge and the resulting products were studied by Wang et al. [70]. A CCP discharge was generated in a stainless-steel cylindrical chamber of 200 mm diameter and 300 mm height in residual air at 15 Pa. The chamber’s temperature was maintained at about 40 °C. The AFB_1_ was exposed to the plasma in powder form after the solutions were dried by nitrogen purging. Various amounts of AFB_1_ (2, 10, and 50 µg) were treated for different lengths of time under several input power conditions (100, 200, and 300 W). Following the plasma treatments, the samples were dissolved in acetonitrile, and their AFB_1_ content was determined with the HPLC method. It was found that independently of the initial AFB_1_ concentration, the 10 min treatment with the 300 W discharge led to a degradation rate of up to 88.3%. The degradation products were identified with ultra-high performance liquid chromatography with quadrupole time-of-flight mass spectrometry (UPLC-Q-TOF MS). Five major compounds were identified (C_12_H_14_O_4_, C_16_H_17_O_9_, C_16_H_17_O_7_, C_17_H_17_O_9_, and C_16_H_17_O_8_), which lost their double bonds in the terminal furan ring, due to which they theoretically had reduced toxicity compared with AFB_1_, according to the structure–activity relationship; albeit, no cell tests were conducted.

Siciliano et al. studied the degradation of four aflatoxins (aflatoxins B_1_, G_1_, B_2_, and G_2_) in standard solutions using an atmospheric pressure DBD [71], which had a coaxial electrode system and operated at about 7 bar in a crosswise gas flow mode. The gas flow rate used was about 120 L/min. The plasma was generated in pure N_2_ and N_2_/O_2_ gas mixtures with a 100–150 kHz voltage. Different powers in the 400−1150 W range and treatment times from 1 to 12 min were tested when the samples were positioned at 50 mm from the plasma source. The treatments were performed in an isolated chamber to maintain the sample in a controlled atmosphere and temperature. The mixtures of the aflatoxins’ standards were prepared by diluting the original standards at the final concentration of 10 ng/mL. As the experiments were conducted with aflatoxins in aqueous solutions, the reactive species were those created in the liquid phase by the plasma–liquid interaction, such as OH, O_2_(a), H_2_O_2_, O_3_, NO_2_^−^, NO_3_^−^, and peroxynitrite [72]. The results showed that the greatest detoxification efficacy was obtained with nitrogen or N/20.1%O_2_ initial mixture discharges at 400 W for a 12 min treatment. When increasing the power to 1000 W, the appropriate treatment time decreased to 2 min. We should note that the nitrogen discharge favors the formation of nitrite, nitrate, and peroxynitrite in the aqueous solution [72]. These results indicate that plasma-activated water might hold relevance for seed decontamination.

### 3.3. Decontamination of Artificially Infected Seeds by Discharge Plasmas

Based on the results obtained using aqueous solutions of aflatoxins, Siciliano et al. [71] used the same DBD setup and procedure to treat 40 g of hazelnuts artificially contaminated with aflatoxins. Shell-less raw hazelnuts were sprayed with an aflatoxin solution containing four aflatoxins, obtaining contamination of 20 ng/g for each aflatoxin. The treatments were performed using pure nitrogen as the initial gas. Unlike the aqueous solution, none of the treatments resulted in total detoxification. However, there was a clear trend towards higher detoxification efficacy with increasing treatment time or power. One should note that although the two treatments look similar, the interacting active species are different as the aflatoxins deposited onto the seeds were treated in the gas phase and not in an aqueous environment. When applying atmospheric nitrogen DBD at a 50 mm distance, it is expected to have the N-atoms, N_2_ metastables, and NO molecules as active species due to the oxygen impurities and UV radiation, which are not the most efficient species for degrading aflatoxins. Consequently, the highest detoxification of AFB_1_ and total aflatoxins on hazelnuts, around 70%, was obtained at the highest 1150 W power with the longest (12 min) treatment time. It was also shown that during the most prolonged treatment at the highest power, the temperature increased to a maximum of 59 °C, which is expected not to affect the qualitative properties of hazelnuts. These treatments were considerably milder and less efficient than those of Park et al. with the argon atmospheric pressure microwave discharge [69], where the charged species also participated in the etching process. At the same time, the temperature and UV radiation were also higher.

A low-frequency plasma jet, similar to the microwave plasma jet used by Park et al., was tested by Dasan et al. [73] on artificially infected maize seeds inoculated with aflatoxin-producing *Aspergillus flavus* and *Aspergillus parasiticus*. After inoculation, the artificially contaminated seeds were incubated at 25–28 °C for 18–24 h to enable mold spores to adhere to the surface and to remove the extra moisture gained during fungal inoculation. The plasma source used was an atmospheric pressure plasma jet (Plasmatreat GmbH, Steinhagen, Germany) equipped with a stainless-steel nozzle. The discharge was generated between the tip of a high-voltage needle electrode (powered with 5–10 kV at 18–25 kHz frequencies, to a maximum power of 655 W) to the inner wall of the nozzle, using a 3000 L/h gas flow rate, due to which the plasma expanded 20 mm outside of the nozzle. The plasma jet was connected to the bottom of the treatment reactors (fluidized bed reactors with diameters of 49 and 65 mm and lengths of 147 and 195 mm, respectively), where the samples were repeatedly conveyed to the treatment zone by employing an aeration gas flow supplied from a compressor with a 900 L/min air yield. As a consequence, the seeds in the reactor were in contact with the plasma afterglow. Forty grams of seeds was treated with dry and filtered air and nitrogen for 1–5 min. It was found that after 5 min of air plasma treatment, the initial fungal load concentration (7 log (cfu/g)) had dropped by 5.48 and 5.20 log (cfu/g) for the *Aspergillus flavus* and *Aspergillus parasiticus* spores, respectively. At the same time, the native microbial flora of the maize grains, initially about 3 log (cfu/g), had decreased to an undetectable level after 3 min of plasma treatment. Compared to the nitrogen plasma, the air plasma was more efficient due to the appearance of reactive oxygen species, such as O-atoms, ozone, and OH, which directly impact the cells of microorganisms, especially on their outermost membranes made up of lipid bilayers. The plasma treatment’s fungicidal effect was attributed to the destruction of the spore integrity. It was also shown that during the storage of the plasma-treated maize at 25 °C for 30 days, the *Aspergillus* spp. spores’ log reduction was maintained with no occurrence of re-growth.

In contrast to atmospheric pressure plasmas where, as illustrated by the works presented above, only a modest amount of seeds can be treated, low-pressure systems provide larger plasma volumes and allow larger amounts of seeds to be treated. Selcuk et al. [74] tested the inactivation of *Aspergillus* spp. and *Penicillium* spp. fungi artificially inoculated on seed surfaces by air and SF_6_ (which is less relevant for food and feedstuff treatment) plasmas. The plasma system used was an inductively coupled discharge (ICP) generated with 300 W input power at 66.6 Pa in a quartz tube of a 5 mm diameter and 40 mm length by applying a 1 kHz sinusoidal 20 kV voltage. In the center of the discharge tube, 8 g of seeds was placed and periodically rotated to ensure the surface of seeds had the same treatment. The studies were conducted on several seeds: wheat (*Triticum durum*), bean (*Phaseolus vulgaris*), chickpea (*Cicer arietinum* L.), soybean (*Glycine max* cv), barley (*Hordeum vulgare* L. cv.), oat (*Avena sativa*), rye (*Secale cereale*), lentil (*Lens culinaris*), and corn (*Zea mays*). The treatment was established to be the most efficient in the case of contaminated wheat. With 20 min of air plasma treatment, the initial fungus colony of 4.1 × 10^7^ cfu/g decreased to less than 1 × 10^4^ cfu/g, which remained stable over time. Similar results were obtained for the SF_6_ plasma. However, with SF_6_, the first phase of the inactivation process occurred at a higher rate. In the case of corn, only a 1-log reduction in the fungal spore population was achieved. The results indicated a relationship with the effectiveness of the plasma being used. It was also found that the plasma treatment had no significant effect on the germination of wheat and bean seeds. The results also showed that the quality of wheat (wet gluten content, gluten index) and beans (water uptake and cooking parameters) were not or were only marginally affected by the plasma treatment. One should note that in this low-pressure system, the seeds were exposed not only to reactive neutral species but also to energetic charge species, which play a significant role in the etching process.

The efficiency of aflatoxin degradation was studied by Basaran et al. [75] in the case of nuts artificially contaminated with aflatoxin-producing *Aspergillus parasiticus*. It was found that the artificially contaminated hazelnuts (106 conidiospores/g) contained 950 ng/g of total aflatoxins (B_1_, B_2_, G_1_, and G_2_) which, following a 20 min SF_6_ treatment, decreased to 751 ng/g. Furthermore, although the air treatment was less efficient in the microbial reduction than the SF_6_ treatment, it reduced the total aflatoxin to 470 ng/g. This result also shows the importance of oxidative species in aflatoxin degradation.

The reduction in the aflatoxin production of *Aspergillus parasiticus* and *Aspergillus flavus*, inoculated on groundnuts by low-pressure, CCP radio-frequency discharge (CCP RF), was investigated by Devi et al. [76]. The discharge was generated in a glass reactor that was 120 mm high and had a 300 mm internal diameter equipped with two parallel electrodes of a 200 mm diameter placed at a 30 mm distance from each other. The working gas was 45% relative humidity air at 20 Pa pressure, while the input powers were 40 W and 60 W when the applied 13.56 MHz voltages were 1500 and 1950 V, respectively. The treatment of 10 g groundnuts at 40 W and 60 W resulted in a 2-log reduction in the initial spore load of 1.45 × 10^3^ cfu/g within 30 min and 24 min, respectively. A 97.9% and 99.3% reduction in the growth of *Aspergillus parasiticus* and *Aspergillus flavus* was found when treated at 60 W power. Due to the high humidity of the air used, the discharge is expected to provide a high concentration of highly reactive OH molecules, besides the reactive oxygen and nitrogen species supplied by an air plasma. Scanning electron microscopy (SEM) micrographs showed the complete disintegration of the fungal spore membrane due to the electroporation and etching caused by the plasma species. It was shown that the 40 W 15 min and the 60 W 12 min treatments reduced the original 0.35 µg/kg AFB_1_ content of *Aspergillus parasiticus*-infected nuts by 70% and 90%, respectively. Using the same treatments, in the *Aspergillus flavus*-infected nuts case, the original 9.84 µg/kg AFB_1_ content was reduced by 65% and 95%, respectively.

A new type of DBD of a large electrode gap designed around a polypropylene box was tested by Shi et al. [77,78] for different humidity air-like N_2_/O_2_ and O_2_/CO_2_/N_2_ mixtures on plated aflatoxins and artificially aflatoxin-contaminated corn. The dimension of the box was 4.4 cm × 18.4 cm × 27.9 cm (H × W × L), and the two disc-shaped 158 mm diameter electrodes were placed on its top and bottom. The discharge was operated in 78% N_2_/22% O_2_ (air) and 65% O_2_/30% CO_2_/5% N_2_ (modified air-MA) at 200 W and 50 Hz with 90 kV voltage between the electrodes. Twenty-five grams of AFB_1_ spiked corn was treated with gases of relative humidity (RH) 5%, 40%, and 80%, respectively, for 1 min to 30 min, in the direct plasma or outside the direct plasma zone in the sealed box. The main species followed were ozone, OH, and NO_x_, which are believed to contribute to aflatoxin degradation. It was found that increasing the relative humidity resulted in a decrease in the ozone concentration in both types of gases. The ozone concentration reached a saturation level at about 10 min of discharge operation, namely at 5% RH 17,500 ppm and 5250 ppm in the case of MA and air, respectively. Similar behavior was also observed for the NO_x_ concentration that reached 12,250 ppm and 1650 ppm, respectively. Higher aflatoxin degradation was found at higher humidity, although the difference between the 40% and 80% RH gases was minimal. The initial aflatoxin level of 420 ng/g after 1 min and 10 min treatment with 40% RH air decreased by 62% and 82%, respectively. Slightly higher degradation was obtained using the MA gas mixture due to the higher NO_x_ and ozone concentrations. The increase in the degradation efficiency with humidity was attributed to the creation of OH molecules in the humid gas discharge. A similar effect was observed in the case of ozonation by McDonough when using a humidified ozone [79]. The maximum 90% percentage of aflatoxin degradation was obtained with a 30 min treatment. It was established that stirring the seeds during treatment could significantly increase the efficiency of degradation, as could storing the seeds in the afterglow plasma for at least 24 h. However, even these conditions could not achieve the 100% aflatoxin decontamination of corn, suggesting that aflatoxin was also present within the kernel, which the plasma species could not reach. Moreover, no difference between the direct and indirect plasma treatments was found, suggesting the charged species did not play an essential role in the aflatoxin degradation. We note that in the case of artificially contaminated seeds, the aflatoxin contamination mainly occurs on the seeds’ surface; yet, in naturally contaminated seeds the inside of the kernel is also infected.

Shi et al. [78] identified the degradation products of aflatoxins treated with the large gap DBD when treating AFB_1_ powder on a glass slide. The degradation products were identified by high performance liquid chromatography with time-of-flight mass spectrometry (HPLC-TOF-MS), and their structures were clarified via orbitrap mass spectrometry through fragmentation of the parental ions. Six main degradation products were observed: C_16_H_16_O_6_, C_17_H_14_O_7_, C_14_H_12_O_5_, C_14_H_10_O_6_, C_17_H_12_O_7_, and C_19_H_18_O_8_, which are different to those found by Wang et al. [70] with the treatment involving low-pressure CCP. Two of these degradation products, C_16_H_16_O_6_ and C_17_H_14_O_7_, were also identified as the products of AFB_1_ ozonolysis [80], suggesting that ozone is one of the main reactive species in this process. Overall, two degradation pathways of AFB_1_ by DBD treatment were proposed: one involving reactions with H, OH, and CHO radicals additions, the other involving epoxidation by HO_2_ radicals and oxidation by the combined effects of the oxidative species OH, H_2_O_2_, and O_3_. It was suggested that the bioactivity of the plasma-treated AFB_1_ was significantly reduced following the disappearance of the C8-C9 double bond in the furofuran ring in all of the major degradation products, as well as the modification of the lactone ring, cyclopentanone, and the methoxyl group.

Table 1 presents a list of heavy molecules observed by different authors after AFB_1_ treatment with gaseous plasma. Many lighter molecules are volatile and have not been probed. The observed modifications are worthy of discussion given the general literature on plasma-surface engineering. First, it should be stressed that plasma treatments mainly cause modification of the surface’s composition and structure. The same mechanism limits any bulk effects, as in the case of ozonation or ammoniation, diffusion. As long as the toxins are present on surfaces only, the interaction between the reactive plasma species and the organic material is extensive. Still, according to the relevant literature, the toxins may also be present inside the grains. The mold can attack kernels during all stages, from silking to maturity, preferentially colonizing the oil-rich germ tissue [81]. Therefore, plasma treatment is more efficient for destroying toxins synthesized by mold during improper storage rather than growth in the field. The concentration of toxins varies significantly among different constituents of a kernel. In one study, AFB_1_ was predominantly found in (or on the surface of) bran, where the concentration was ten times higher than the average of the corn seed [82]. In such cases, plasma treatment may be more efficient than other methods for destroying toxins.

Any interaction of gaseous plasma with organic molecules will bring at least two effects: (i) surface functionalization and (ii) etching. Besides, the UV and VUV radiation, or both, will cause structural modification of organic material through radiation damage [83].

The surface functionalization upon the treatment of organic material with a plasma sustained in a gas containing oxygen (such as air) occurs through the chemical bonding of reactive oxygen species to the carbon atoms of the organic matter. The functionalization leads to enrichment of the organic material surface with oxygen. With regard to Table 1, the functionalization of aflatoxin by oxygen plasma treatment leads to partial functionalization with oxygen as products containing the same amount of carbon atoms (17 in the case of AFB_1_) contain more oxygen (7 or even 9, compared to 6 for untreated AFB_1_). Apart from compounds containing 17 C-atoms, Table 1 also reveals several chemicals with 16 C-atoms only. The concentration of oxygen in these chemicals varies but is larger than the concentration in the untreated AFB_1_. The results summarized in Table 1 therefore confirm the functionalization of the original toxin with oxygen. Products with fewer carbon atoms were also detected, as shown in Table 1. Such products may be the remains of incomplete oxidation.

The etching of organic material coincides with surface activation. Many authors have determined the etching rates for simple organic matter, with the results varying. A typical etching rate for many polymers at room temperature is often of the order of nanometers per second [84]. The etching of organic matter sustained at the floating potential in weakly-ionized highly reactive oxygen plasma is often explained by the complete oxidation of organic material upon treatment with plasma rich in reactive oxygen species. The reaction products of complete oxidation are CO_2_ and H_2_O. These molecules are volatile and were not probed by the authors who reported that the toxins had been destroyed by gaseous plasma treatment. The technique was commercialized decades ago and is often called “plasma degreasing”, “plasma stripping”, “plasma ashing”, or “discharge cleaning”. Whatever the nomenclature, the oxygen plasma treatment was confirmed as an effective method for removing organic matter by oxidation. Yet, the oxidation might not always be complete. An intermediate stage involves the forming of various oxidized organic compounds that may be unstable and thus decay spontaneously well after the plasma treatment has been accomplished. The results in Table 1 reveal such incomplete oxidation: the formation of organic molecules with less carbon content than in the original toxin. Other molecules, such as aldehydes, ketones, and acids, are probably also formed upon the treatment of toxins but have not been probed by the authors of the scientific literature concerning the treatment of toxins by gaseous plasma. The science of plasma-toxin interaction is, therefore, still in its infancy, although the results summarized in this review hold promise with respect to the future application of this technique for purifying crops contaminated with toxins. The exact mechanisms at work in the plasma-oxidation of toxins are currently unknown and constitute a huge, tremendous scientific challenge.

Table 2 provides a summary of the gas discharge plasma decontamination of seeds from aflatoxin B_1_ and *Aspergillus* fungi.

## 4. Main Points and Future Perspectives

Although the methods for destroying aflatoxins have been reviewed in this paper, different authors have unfortunately used different experimental setups, making the results hardly comparable. Still, one can draw the following conclusions:

1. The destruction of toxins remains both a scientific and technological challenge.
2. The reported treatment times are prohibitively long for industrial application.
3. The incomplete description of the particularities of the experimental setups and treatment conditions prevents valuable conclusions from being drawn.
4. The plasma methods seem promising, but upscaling poses a significant challenge.

**Figure 5:**
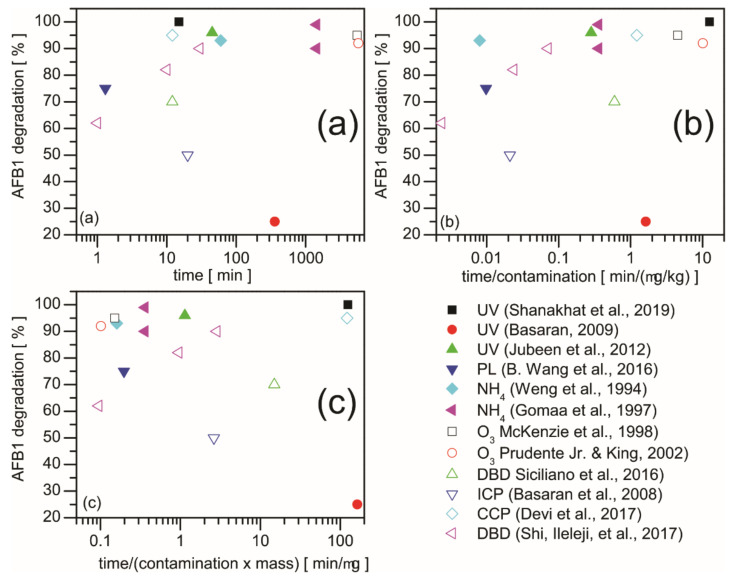
AFB_1_ degradation efficiency versus (**a**) treatment time in min, (**b**) time/contamination in min/(mg/kg), and (**c**) time/contamination × mass) in min/kg achieved with different methods and contaminated samples. UV and pulsed light treatments are presented for artificially contaminated 100 g of semolina [19], 8 g of hazelnut [20], 250 g of nuts [21], and 50 g of rice [23]. Ammoniation experiments were conducted on 50 g of grounded naturally contaminated corn [29] and 1 kg of artificially infected corn [30]. The greatest efficiency was achieved with the high-pressure high-temperature (HP/HT) method. Ozonation results are presented for 30 kg [32] and 100 kg [35] of naturally contaminated corn. The gas discharge plasma treatments were performed with 40 g of hazelnut [71], 8 g of hazelnut [75], 10 g of groundnut [76], and 25 g of corn [77]. For the treatment details, see the related sections in this paper.

Despite the incomparability of the different authors’ results, we present the correlations in Figure 5. Three diagrams are plotted for the AFB_1_ decontamination: the reported degradation efficiency (i) versus treatment time (Figure 5a), (ii) versus treatment time divided by the contamination rate (Figure 5b), and (iii) versus treatment time divided by the product of the contamination rate and mass of the sample (Figure 5c).

While different pathways of toxin destruction have been proposed and evaluated by several authors, it is generally concluded that the chemical reactions mainly occur on the surface of the material composed of or containing toxins as the diffusion of active species into the bulk material is limited. Furthermore, the rates of the chemical reactions increase with temperature, while the detoxification rates depend on the flux of reactants onto the surface. From this point of view, the high-pressure gaseous treatments at elevated temperatures are preferable. Such treatments are, of course, limited to the heat the seeds can withstand. A good example is ammoniation, where efficiency increases with pressure and temperature. Although the treatment times are long (see Figure 5a), this method can be applied in the case of large storage containers where long treatment times (measured in days, even weeks) are feasible to allow for the diffusion of the ammonia (Figure 5c).

The ozonation is also limited by diffusion (requiring long treatment times; Figure 5a). However, in this case the degradation results in volatile products, which ensure the low reversibility of the process. The oxidation of toxins is therefore more efficient than ammoniation. Yet, the available ozone concentration limits the flux of ozone molecules onto the surface of samples contaminated with toxins. Another problem with ozonation is poor selectivity: the ozone molecules will not only interact with the toxins but with many other materials, including the grains themselves. This means that the expenditure for ozone production may be too high for commercial applications.

Ultraviolet radiation remains a promising technique for the destruction of toxins, but the relevant literature does not reveal the influence of photon energy. Photons are likely to penetrate quite deep into the organic matter and thus this technique is not limited to surface effects. The penetration depth depends enormously on the photon energy (or the UV radiation wavelength). Low-energy UV photons (such as those from medium-pressure mercury lamps) penetrate deep into the organic matter, stimulate photo-oxidation, photo-degradation, and photo-elimination (or both), which was found beneficial for the degradation of aflatoxins. Not much research has been performed on the degradation of toxins using high-energy photons.

Gas discharge plasmas hold considerable potential for detoxifying agricultural products. Plasmas contain reactive species of oxidation potential exceeding the potential of ozone and may also be a significant source of UV radiation, depending on the discharge configuration. Both low-pressure and atmospheric discharges were used for the destruction of toxins. Unfortunately, the authors did not report the fluxes of reactive plasma species of high oxidation potential onto the sample surfaces, meaning it is impossible to compare their results with those obtained for ozone. The reactive species with high oxidation potential likely to be found in gaseous plasma sustained in the air include the O-atoms (in both the ground and metastable states), the NO molecules, and the N-atoms. Their density in non-equilibrium gas discharge plasmas is limited either by gas-phase or surface effects. Still, their production is energetically beneficial as, in such discharges, a significant part of the available discharge power is spent on dissociation. While the relatively short treatment times needed for efficient detoxification using gas discharge plasma (Figure 5) are encouraging, more systematic work with well-characterized plasmas should be performed. The available scientific works on plasma techniques reviewed in this paper are limited to academic research using a small mass of samples and relatively weak contamination. This means the systems need to be scaled up to make the plasma technique competitive.

## Figures and Tables

**Table 1:** Heavy molecules observed upon the treatment of aflatoxin B_1_ (C_17_H_12_O_6_) by discharge plasma.

C_19_H_18_O_8_			
C_17_H_17_O_9_	C_17_H_12_O_7_	C_17_H_14_O_7_	
C_16_H_17_O_9_	C_16_H_17_O_7_	C_16_H_17_O_8_	C_16_H_16_O_6_
C_14_H_12_O_5_	C_14_H_10_O_6_		
C_12_H_14_O_4_			

**Table 2:** Summary of the gas discharge plasma decontamination of seeds from aflatoxin B1 and Aspergillus fungi.

System	Inoculated Seeds	Quantity	Treatment Time	Input Power	Initial AFB_1_ Concentration	Reduction
N_2_ DBD [71]	hazelnut	40 g	12 min	1150 W	20 ng/g	70%
air ICP [75]	hazelnut	8 g	20 min	300 W	950 ng/g	50%
45%RH air CCP [76]	groundnuts	10 g	12 min	60 W	9.84 ng/g	95%
40%RH air DBD [77]	corn	25 g	1 min	200 W	420 ng/g	62%
			10 min			82%
			30 min			90%
air RF jet [73]	maize	40 g	5 min	655 W	10^7^ cfu/g	5 log
air ICP [74]	maize	8 g	20 min	300 W	5 × 10^6^ cfu/g	1 log
45%RH air CCP [76]	groundnuts	10 g	24 min	60 W	1.45 × 10^3^ cfu/g	2 log
